# The value of MALDI-TOF failure to provide an identification of *Staphylococcal* species direct from blood cultures and rule out *Staphylococcus aureus* bacteraemia: a post-hoc analysis of the RAPIDO trial

**DOI:** 10.1099/acmi.0.000192

**Published:** 2020-12-14

**Authors:** Fergus Hamilton, Rebecca Evans, Alasdair MacGowan

**Affiliations:** ^1^​ Infection Sciences, Pathology, North Bristol NHS Trust, Bristol, UK; ^2^​ Population Health Sciences, University of Bristol, Bristol, UK; ^3^​ Bristol Trials Centre, Bristol Medical School, University of Bristol, Bristol, UK

**Keywords:** *Staphylococcus*, MALDI-TOF, diagnosis, bacteraemia

## Abstract

**Introduction:**

Rapid differentiation between *Staphylococcus aureus* (SA) and coagulase-negative staphylococci (CoNS) is critical in clinical infection. Direct matrix-assisted laser desorption/ionization time-of-flight (MALDI-TOF) identification from blood culture is highly accurate, but is associated with a significant failure rate, delaying identification. However, MALDI-TOF failure may itself be indicative of CoNS infection.

**Aim:**

We sought to examine whether failure of MALDI-TOF direct ID was indicative of CoNS infection and could be used as a diagnostic tool to promote antimicrobial stewardship.

**Methodology:**

Results of Gram stains, MALDI-TOF identification and formal identification were extracted from the large, multi-centre RAPIDO trial. All blood cultures with presumed staphylococci were included. MALDI-TOF performance (correct identification, incorrect identification, failed identification) was calculated for each sample and across sites. Risk of SA disease was calculated for each group (correct, incorrect, failed) and across sites. Logistic regression was used to identify if clinical features are associated with MALDI-TOF performance.

**Results:**

In the RAPIDO trial, 4312 patients were allocated to the MALDI-TOF arm. After exclusions, 880 patients were eligible and had a blood culture with a Gram stain consistent with presumed staphylococci. In total, 204 of these (23.2 %) were ultimately identified as SA. MALDI-ID was successful 83.9 % of the time, and was 100 % accurate when successful. Failure was more common in CoNS isolates (124/641, 19.3 %) than in SA (13/191, 6.4 %). When MALDI-TOF failed, the risk of SA disease was 9.2 % across the whole cohort, although failure rates and risk of SA disease varied significantly between centres. MALDI-TOF failure was independent of clinical characteristics.

**Conclusion:**

Presumed staphylococci that fail direct MALDI-TOF identification from blood culture are significantly more likely to be CoNS isolates than SA. In low-risk or low-prevalence settings, SA therapy can be withheld if MALDI-TOF is unsuccessful.

## Introduction

Matrix-assisted laser desorption/ionization time-of-flight (MALDI-TOF) identification has revolutionized the clinical microbiology laboratory, reducing the usage of complex biochemical testing, and increasing the rapidity of microbial diagnosis, with two large commercial systems available [1, 2]. The RAPIDO trial aimed to identify if there was any value in performing MALDI-TOF direct from blood culture, to facilitate early diagnosis and management of the identified organism. This was a randomized controlled trial (RCT) that compared traditional testing (Gram stain performed on positive blood culture, followed by growth then identification by phenotypic testing or MALDI) versus MALDI-TOF directly from blood culture bottles. This recently published trial showed that direct MALDI did not appear to make an impact on clinical outcomes, although there was a trend for reduced mortality in the subgroup with *Pseudomonas a *e* ruginosa *, often not covered with empirical therapy [3].

Despite this, there is increasing use of MALDI-TOF direct from blood culture, and it is common in many clinical laboratories [2]. Generally, it is reliable from blood culture, with around 80 % of organisms being identified correctly, with the vast majority of these having the ID subsequently confirmed by traditional testing or MALDI-TOF on the cultured isolate [2].

Clinicians who use MALDI-TOF are now faced with another problem: what to do when the isolate fails to identify directly from blood culture? This situation is most common with presumed staphylococci, where the management of *Staphylococcus aureus* (SA) infection is often radically different from other staphylococci, whose prevalence is generally significantly higher. In most cases of SA bloodstream infection, therapy is urgent and mandated for a long period (usually a minimum of 2 weeks of intravenous treatment), whereas with coagulase-negative staphylococci (CoNS), management is not required at all or is much less aggressive [4].

As clinical experience of the direct use of MALDI-TOF has increased, there has been an increasing incidence of this problem. In our laboratory, organisms unable to be directly identified often turned out to be CoNS, although this was purely based on observation.

In this post-hoc analysis of data from the RAPIDO trial, we aimed to see if this observation was true, and whether any useful prognostic information could be gained from isolates that failed to identify via direct MALDI-ID, and whether the absence of a direct MALDI-ID should alter management of presumed staphylococci identified on the Gram stain.

## Methods

### Population

This study undertook post-hoc analyses of data from the RAPIDO trial, and a full protocol of the trial is available with the original publication [3]. Briefly, the RAPIDO trial was a multi-centre RCT that compared direct MALDI-ID from blood culture bottles to traditional management of a positive blood culture. This reduced the time to pathogen identification by 24 h, but did not improve mortality. In the trial, both bottles of a standard blood culture set were used as normal, with MALDI identifications being performed usually only on the first one (as would be standard practice). All MALDI was performed using the Bruker Sepsistyper system.

In this analysis, we aimed to identify all patients with possible staphylococcal infection in the direct MALDI-ID arm, to assess the prognostic value of a failed MALDI identification.

### Outcome measures

The primary outcome for this analysis was the prevalence of SA compared to any other Gram-positive cocci (GPC) using the final identification. The secondary outcome was MALDI-ID failure.

### Statistical analysis

For our primary analysis, we identified all patients who were in the direct MALDI arm, and identified all those whose Gram stain would have been GPC in clusters (i.e. presumed staphylococci), such as *Staphylococcus* or *Microcococcus*. The RAPIDO trial did not record the Gram stain findings in more detail than GPC, so all organisms that would have not had a stain supporting a *Staphylococcus* identification were removed.

Next, we calculated the prevalence of SA as compared to any other GPC using the final identification. This was done for the population as a whole and also separately for each individual site, assuming there may be a different prevalence of SA at each site depending on their patient population. We then identified all bottles that had a failed MALDI-ID, and recorded their final identification. Using this, we were able to calculate a pre-test probability of SA disease, given the presumptive Gram stain, and the post-test probability, given a failed MALDI-ID. This was performed for each site, allowing us to calculate estimates at the site level.

For our secondary analysis, logistic regression was used to investigate whether the MALDI-ID was independent of other predictive factors such as age, white cell count, site, fever and blood pressure. Definitions of these are contained within the full RAPIDO trial protocol [3].

All analysis was performed in R 3.6.1, using the *tidyverse* package.

## Results

In the trial, 8628 of 14 298 positive blood cultures were randomized, with 4312 allocated to the MALDI-TOF arm. After excluding samples that were ineligible, or from patients who declined consent, 2197 participants were eligible for inclusion in this analysis.

In total, 1283/2197 initial MALDI-IDs from the initial bottle had a Gram stain consistent with GPC, and 880 of these were *Staphylococcus* or *Micrococcus* species; a breakdown is presented in Table 1. CoNS were more common than SA (641 vs 204 isolates).

**Table 1.** Breakdown of initial MALDI results, for the three relevant organisms

Final ID (number of cases)	MALDI ID result for each organism		
	Correct (*n*, %)	Incorrect (*n*, %)	Unable to ID (*n*, %)
* Staphylococcus aureus* (204)	191 (93.6 %)	0 (0.0 %)	13 (6.4 %)
CoNS (641)	517 (80.7 %)	0 (0.0 %)	124 (19.3 %)
* Micrococcus* (35)	30 (85.7 %)	0 (0.0 %)	5 (14.3 %)
**Overall (880**)	**738 (83.9 %)**	**0 (0.0 %)**	**142 (16.1 %)**

MALDI-ID was 100 % accurate when performed, with no isolate incorrectly identified. However, failure to ID occurred in (142) 16 % of the participants. SA was identified correctly by MALDI 93.6 % of the time, whereas both CoNS and *Micrococcus* were less accurately identified (80.7 and 85.7 % respectively).

### Pre- and post-test probabilities

CoNS isolates and *Micrococcus* species were much more likely to have an inconclusive direct MALDI-ID. As such, the presence of an inconclusive direct MALDI-ID increased the probability of the isolate being CoNS or *Micrococcus *, based on Bayes’ rule.

In the study as a whole, 23.2 % of included GPCs were SA, but this value was only 9.2 % of those with a failed ID. In other words, the probability of any given isolate with a Gram stain showing GPC being SA was 23.2 % before the MALDI-ID was performed, and 9.2 % if the MALDI was performed and failed. In contrast, the probability of an isolate being a CoNS or *Micrococcus* was 76.8 % before the MALDI-ID and 90.8 % if the MALDI-ID failed.

### Performance between centres

Across the seven sites in the MALDI-TOF trial, there was variation in the prevalence of SA disease, with the lowest prevalence at site 2 (18.9 %) and the highest at site 4 (28.6 %) but also variation in MALDI-TOF performance, with failure rates of 6.4 % at site 2 and 34.6 % at site 6 (Table 2).

**Table 2.** Differential prevalence of SA, and performance of MALDI, across the sites

Site (no. of GPC isolates)	No. of GPC that were SA (%, *n*)	Failure rate of MALDI-IDs (%, *n*)	Post-test probability of SA (failed MALDI ID)
1 (*n*=142)	20.4 % (29)	21.8 % (31)	12.9 % (4/31)
2 (*n*=170)	18.9 % (32)	6.4 % (11)	0 % (0/11)
3 (*n*=231)	23.4 % (54)	13.4 % (31)	9.7 % (3/31)
4 (*n*=49)	28.6 % (14)	14.2 % (7)	0 % (0/7)
5 (*n*=174)	27.0 % (47)	13.7 % (24)	16.7 % (4/24)
6 (*n*=49)	22.4 % (11)	34.6 % (17)	11.8 % (2/17)
7 (*n*=65)	26.2 % (17)	32.3 % (21)	0 % (0/21)

At three sites, (2, 4 and 7), no single failed MALDI ID subsequently turned out to have SA, despite sites 4 and 7 having a high prevalence of SA.

### Logistic regression

Table 3 gives the baseline clinical characteristics of those with and without a failed MALDI-ID.

The results of the logistic regression model (with failed MALDI-ID as the outcome variable) are reported in Table 4. This showed that no clinical features were significantly associated with the MALDI result. Unsurprisingly, given the result above, certain sites were significantly associated with the performance of MALDI-TOF.

**Table 3.** Basic clinical demographics of patients with successful and failed MALDI-IDs

Variable	MALDI-ID successful	MALDI-ID failed
*n*	739	141
Age (years; median, interquartile range, IQR)	69 (53, 80)	69 (56, 81)
Gender (% male)	387 (52 %)	86 (61 %)
On immunosuppressive drugs at time 0 (%)	75 (11 %)	19 (15 %)
Missing	53	11
On chemotherapy (%)	69 (9.8 %)	7 (5.3 %)
Missing	36	9
On ventilation at day 0 (%)	77 (11 %)	18 (14 %)
Missing	49	10
On vasopressors at day 0 (%)	56 (8.2 %)	19 (15 %)
Missing	55	14
Neutrophil count (×10^6^/µl at day 0; median, IQR)	8.4 (5.3, 12.3)	9.5 (6.7, 14.2)
Missing	43	14
Temperature (°C) on day 0 (median, IQR)	38.00 (37.10, 38.50)	37.90 (37.00, 38.40)
Missing	71	15
Systolic blood pressure on day 0 (mean, sd)	126 (109, 145)	124 (111, 141)
Missing	101	19

**Table 4.** Multivariable regression with failed MALDI-ID as the outcome variable

Variable	Odds ratio	(95 % confidence interval)	*p*-value
Age	0.99	(0.97, 1.00)	0.225
Gender (male)	0.78	(0.51, 1.19)	0.260
Chemotherapy in last month	2.67	(0.95, 9.61)	0.087
Neutrophil count at day 0 or closest	0.99	(0.95, 1.02)	0.411
On ventilation at day 0	1.16	(0.58, 2.42)	0.687
Systolic blood pressure at day 0 or closest	1.00	(0.99,1.01)	0.81
On vasopressor drugs at day 0	0.53	(0.26,1.08)	0.07
On immunosuppressive drugs at time 0	0.65	(0.34,1.29)	0.20
Centre (largest as reference)			
3	*Reference*		
1	0.61	(0.32, 1.12)	0.11
2	2.04	(0.96, 4.65)	0.07
4	0.84	(0.31, 2.70)	0.75
5	1.15	(0.57, 2.36)	0.69
6	0.302	(0.13, 0.69)	0.004
7	0.405	(0.19, 0.85)	0.001

## Discussion

This paper shows failed MALDI-ID on a blood culture with a Gram stain showing GPC is suggestive that SA is unlikely to explain the result. Only 13 out of 142 failed MALDI-IDs were subsequently identified as SA, with a baseline rate of SA disease being around one-quarter. In three centres from this study, no single failed ID subsequently turned out to be SA.

This suggests that a failed MALDI-ID is generally helpful, and adds significant prognostic information, reducing the probability of SA disease from 23.2 to 9.2 % across the whole study. Importantly, the value of this depends on two factors: local SA prevalence, which affects the base ‘risk’ of SA disease, and local performance of MALDI-ID.

It is also clear that clinical and real-life MALDI-ID performance is dependent both upon the technical skills of the operators (with large differences between centres), but also upon other unknown factors, impacting significantly on interpretability. For example, site 7 had a high prevalence of SA disease (26.2 %), and very high rate of MALDI-ID failure (32.3 %), but none of these failed IDs were identified as SA. In contrast, site 6 had a slightly lower prevalence of SA disease, an even higher rate of MALDI-ID failure, and 11.8 % of these were subsequently identified as SA. The reasons for this discrepancy deserve exploring in further research, and explorations of potential options. It may be that this simply reflects weakness of the database developed by Bruker, or something specific about CoNS isolates that make them hard to identify.

### Strengths and weaknesses

The strength of this study lies in robust data collection (as a subgroup of an RCT) and large numbers, so confident conclusions can be drawn. However, the performance of MALDI-ID in real life may well be different from the RCT setting, and clearly varies by site. Interpretation of this paper will require local knowledge of SA prevalence, and of MALDI-ID failure rates. We only evaluated the Bruker Sepsityper system, as this was used in the RAPIDO trial, and we cannot comment on other MALDI-ID systems.

### Comparisons with other literature

Previous research has shown the value of MALDI-TOF in direct identification of blood cultures, showing a significant decrease in time to identification in RAPIDO [3], and with observational data supporting the accuracy and timeliness of direct MALDI-TOF [1, 5–9]. Recent work on SA has shown the potential for rapid identification of methicillin resistance using a directly targeted assay with cefoxitin added to the droplets and incubated before addition of matrix [10].

There have been two relevant RCTs performed, RAPIDO in the UK and MALDITOF in Vietnam. RAPIDO included mortality as an endpoint, and showed no difference in patient mortality despite an impressive reduction in time to appropriate identification from blood sampling (median time to microbial identification: 38.5 h in the MALDI arm, 50.3 h in the conventional arm) [3]. In MALDITOF, the primary outcome was appropriateness of antibiotic therapy at 24 h; there was no difference found between either arm (41.4 % MALDI arm vs 39.7 % control arm, adjusted odds ratio 1.17, *P*=0.40). In exploratory analyses, there was also no difference in mortality between the two arms (16 % MALDI arm vs 14.2 % control arm, adjusted odds ratio 1.13, *P*=0.59) [7]. The results of these studies suggest that there is limited mortality benefit from the use of MALDI-TOF, although there are clear potential advantages in laboratory processing, efficiency and practicality.

However, as far as we are aware, no previous study has identified features suggesting SA disease from a failed MALDI-ID, or utilized the failure of MALDI-ID as a diagnostic test in of itself, with most noting that MALDI-ID is simply reliable when it is successful.

## Conclusion

A failed MALDI-ID is still useful. Most isolates which failed MALDI-ID were not SA, with the vast majority being CoNS. Although the prevalence of SA disease in this study was 23.2 %, only 9.2 % of isolates that failed a MALDI-ID were subsequently identified as SA. Clinicians should utilize this information in clinical decisions, and should consider holding treatment for SA in patients with low pre-test probability of disease, and a failed MALDI-ID.
